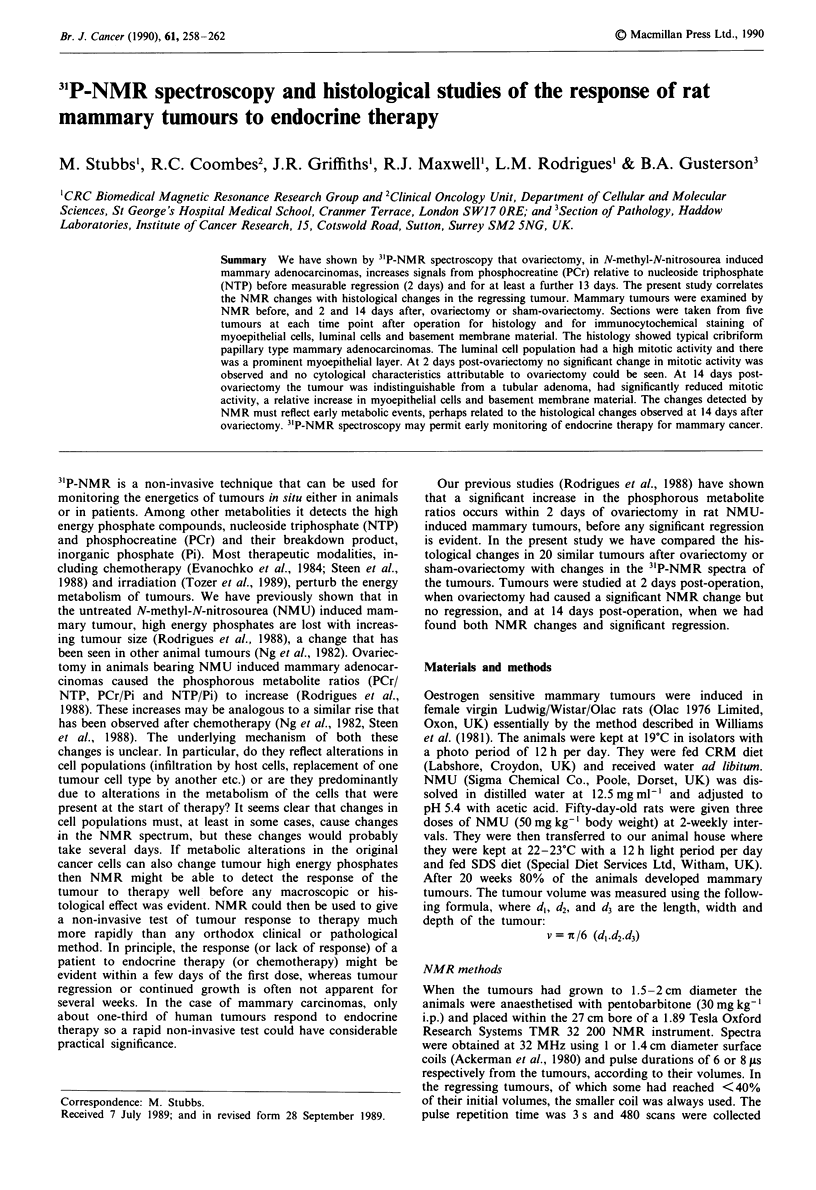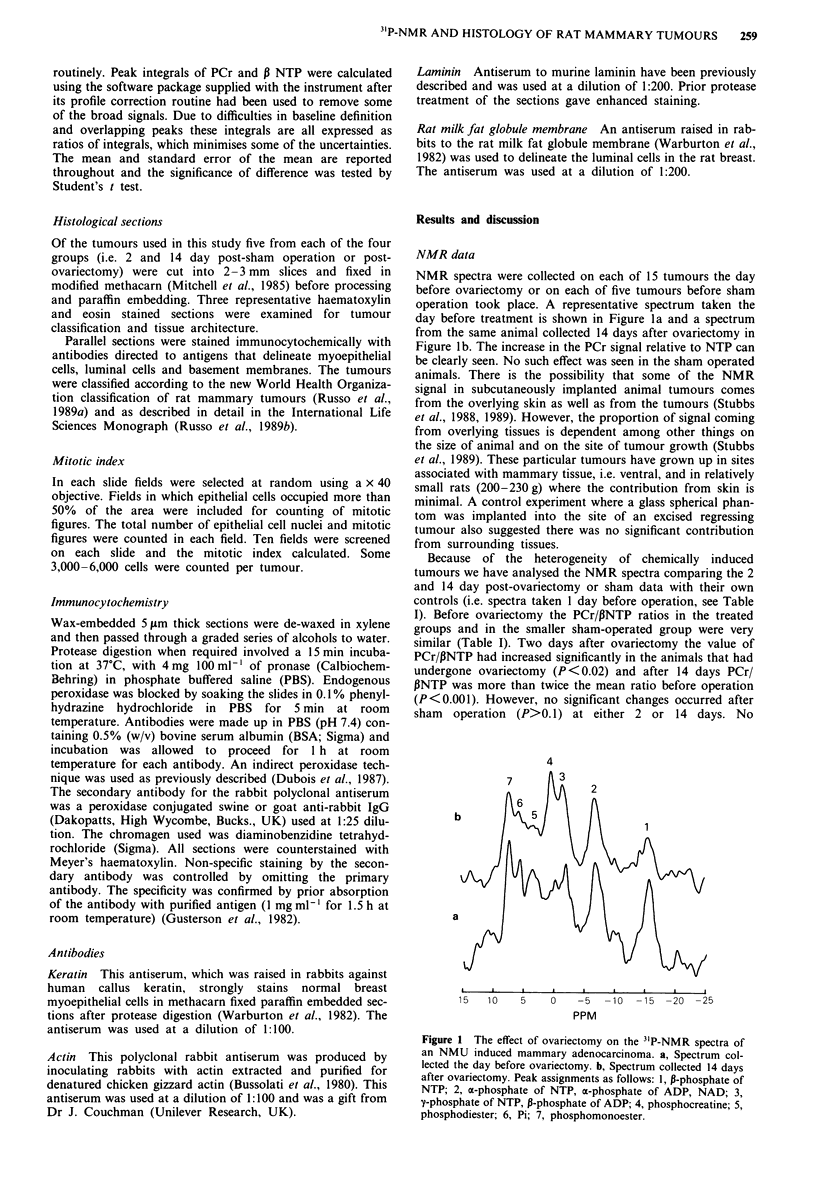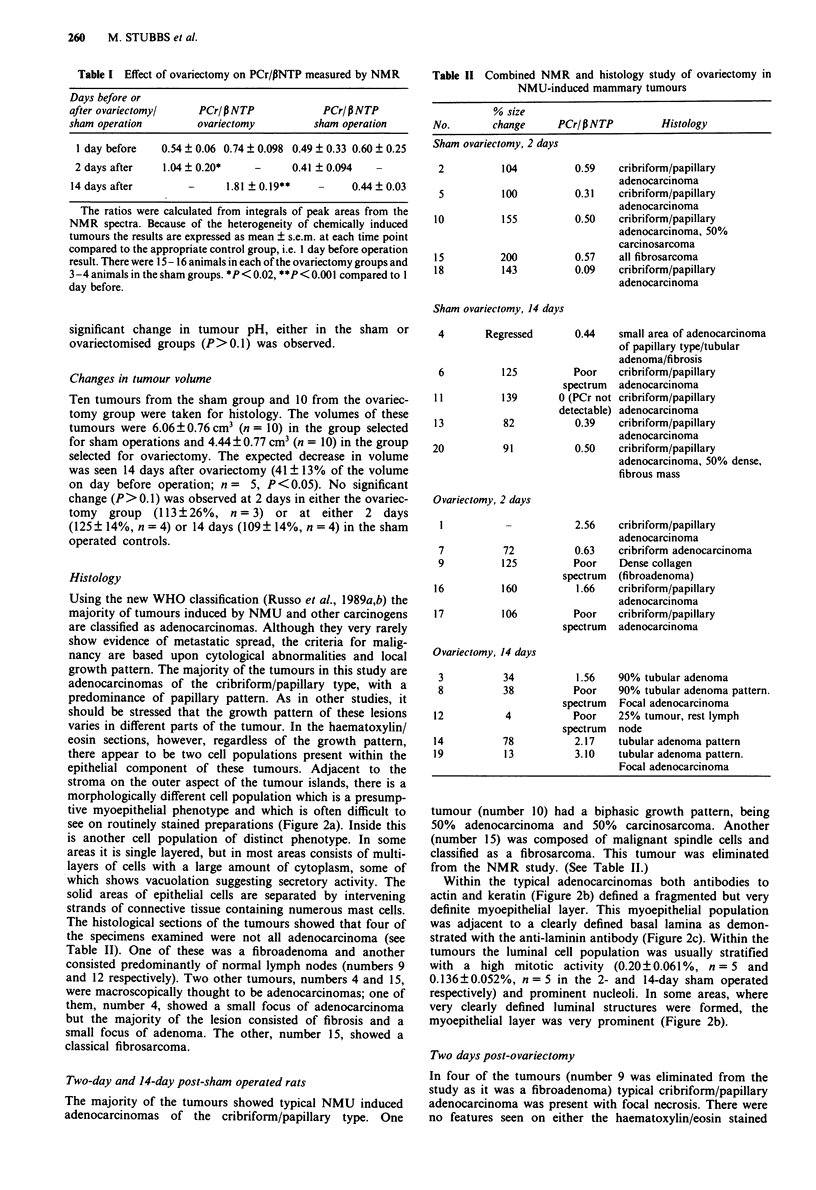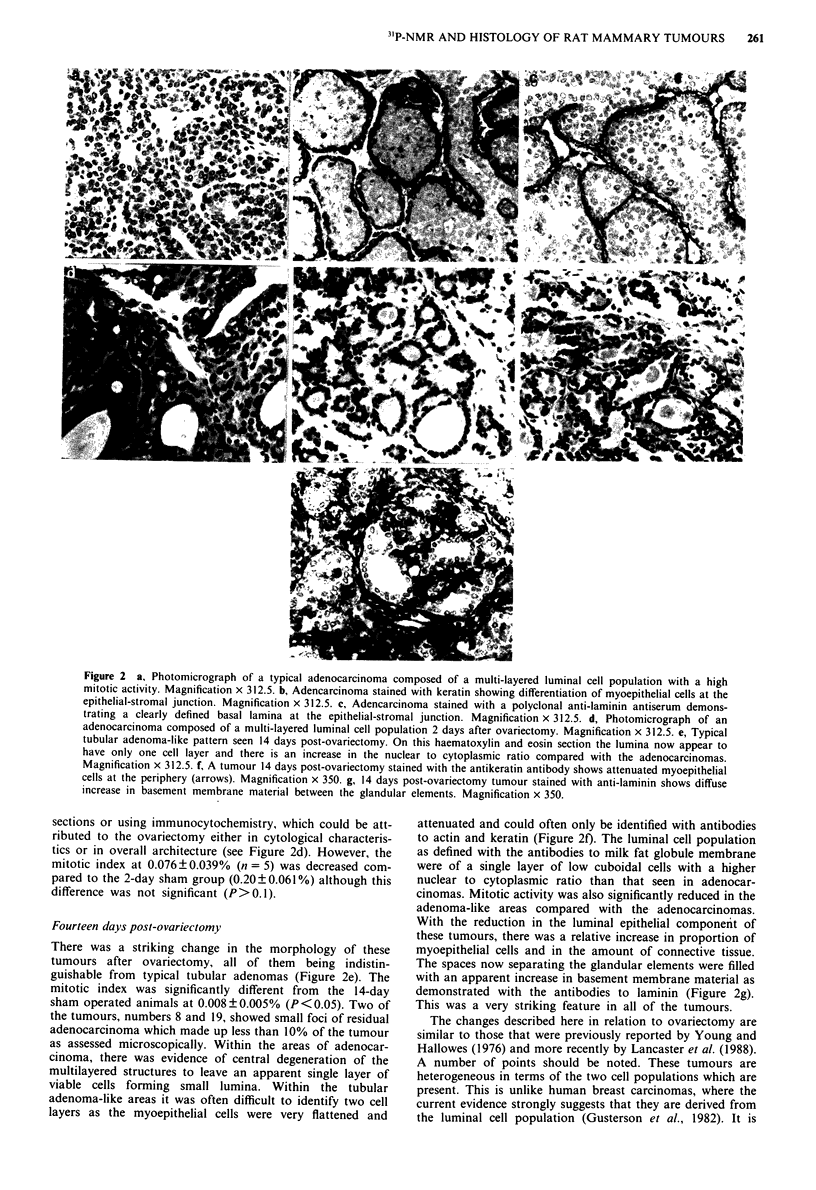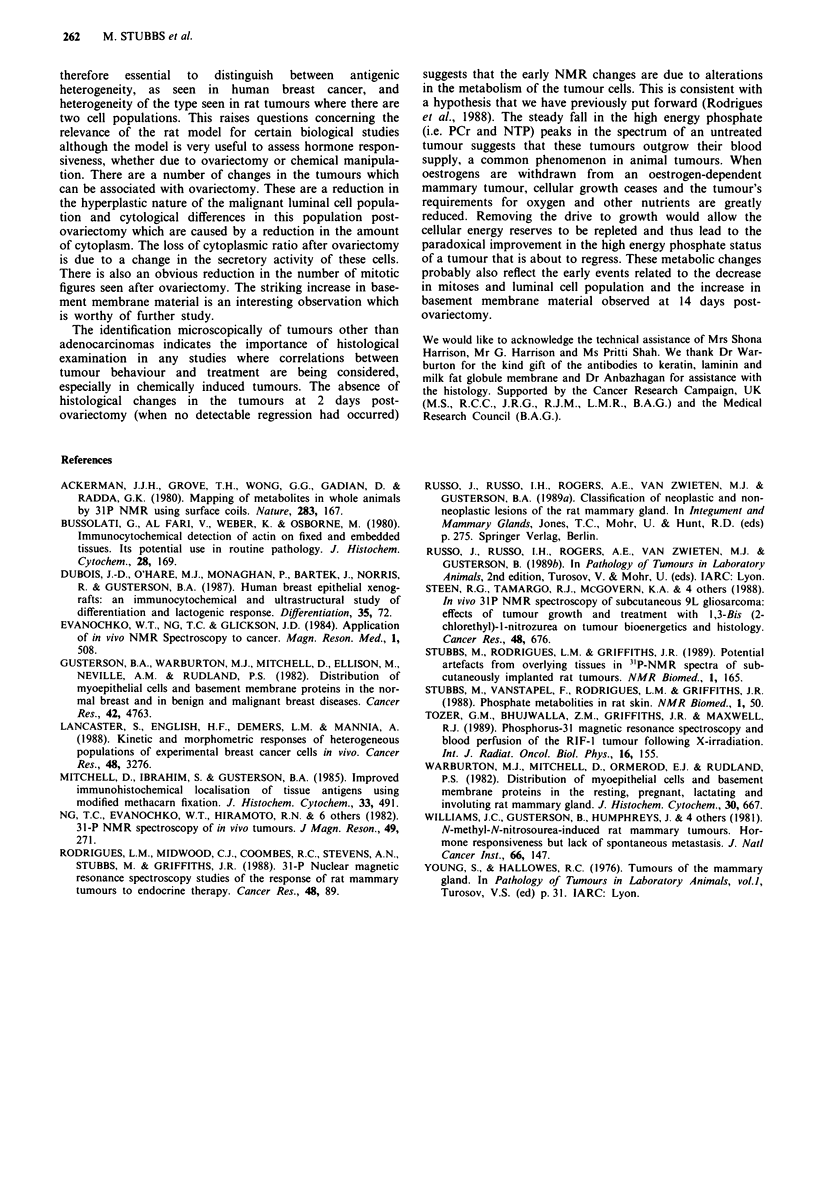# 31P-NMR spectroscopy and histological studies of the response of rat mammary tumours to endocrine therapy.

**DOI:** 10.1038/bjc.1990.47

**Published:** 1990-02

**Authors:** M. Stubbs, R. C. Coombes, J. R. Griffiths, R. J. Maxwell, L. M. Rodrigues, B. A. Gusterson

**Affiliations:** CRC Biomedical Magnetic Resonance Research Group, St George's Hospital Medical School, London, UK.

## Abstract

**Images:**


Br. J. Cancer (1990), 61, 258-262 © Macmillan Press Ltd., 1990

### [31]P-NMR spectroscopy and histological studies of the response of rat mammary tumours to endocrine therapy

M. Stubbs[1], R.C. Coombes[2], J.R. Griffiths[1], R.J. Maxwell[1], L.M. Rodrigues[1] & B.A. Gusterson[3]

[1]*CRC Biomedical Magnetic Resonance Research Group and* [2]*Clinical Oncology Unit, Department of Cellular and Molecular Sciences, St George's Hospital Medical School, Cranmer Terrace, London SW17 0RE; and* [3]*Section of Pathology, Haddow Laboratories, Institute of Cancer Research, 15, Cotswold Road, Sutton, Surrey SM2 5NG, UK.*

**Summary** We have shown by [31]P-NMR spectroscopy that ovariectomy, in *N*-methyl-*N*-nitrosourea induced mammary adenocarcinomas, increases signals from phosphocreatine (PCr) relative to nucleoside triphosphate (NTP) before measurable regression (2 days) and for at least a further 13 days. The present study correlates the NMR changes with histological changes in the regressing tumour. Mammary tumours were examined by NMR before, and 2 and 14 days after, ovariectomy or sham-ovariectomy. Sections were taken from five tumours at each time point after operation for histology and for immunocytochemical staining of myoepithelial cells, luminal cells and basement membrane material. The histology showed typical cribriform papillary type mammary adenocarcinomas. The luminal cell population had a high mitotic activity and there was a prominent myoepithelial layer. At 2 days post-ovariectomy no significant change in mitotic activity was observed and no cytological characteristics attributable to ovariectomy could be seen. At 14 days post-ovariectomy the tumour was indistinguishable from a tubular adenoma, had significantly reduced mitotic activity, a relative increase in myoepithelial cells and basement membrane material. The changes detected by NMR must reflect early metabolic events, perhaps related to the histological changes observed at 14 days after ovariectomy. [31]P-NMR spectroscopy may permit early monitoring of endocrine therapy for mammary cancer.

[31]P-NMR is a non-invasive technique that can be used for monitoring the energetics of tumours *in situ* either in animals or in patients. Among other metabolities it detects the high energy phosphate compounds, nucleoside triphosphate (NTP) and phosphocreatine (PCr) and their breakdown product, inorganic phosphate (Pi). Most therapeutic modalities, including chemotherapy (Evanochko *et al*., 1984; Steen *et al*., 1988) and irradiation (Tozer *et al*., 1989), perturb the energy metabolism of tumours. We have previously shown that in the untreated *N*-methyl-*N*-nitrosourea (NMU) induced mammary tumour, high energy phosphates are lost with increasing tumour size (Rodrigues *et al*., 1988), a change that has been seen in other animal tumours (Ng *et al*., 1982). Ovariectomy in animals bearing NMU induced mammary adenocarcinomas caused the phosphorous metabolite ratios (PCr/NTP, PCr/Pi and NTP/Pi) to increase (Rodrigues *et al*., 1988). These increases may be analogous to a similar rise that has been observed after chemotherapy (Ng *et al*., 1982, Steen *et al*., 1988). The underlying mechanism of both these changes is unclear. In particular, do they reflect alterations in cell populations (infiltration by host cells, replacement of one tumour cell type by another etc.) or are they predominantly due to alterations in the metabolism of the cells that were present at the start of therapy? It seems clear that changes in cell populations must, at least in some cases, cause changes in the NMR spectrum, but these changes would probably take several days. If metabolic alterations in the original cancer cells can also change tumour high energy phosphates then NMR might be able to detect the response of the tumour to therapy well before any macroscopic or histological effect was evident. NMR could then be used to give a non-invasive test of tumour response to therapy much more rapidly than any orthodox clinical or pathological method. In principle, the response (or lack of response) of a patient to endocrine therapy (or chemotherapy) might be evident within a few days of the first dose, whereas tumour regression or continued growth is often not apparent for several weeks. In the case of mammary carcinomas, only about one-third of human tumours respond to endocrine therapy so a rapid non-invasive test could have considerable practical significance.

Correspondence: M. Stubbs.
Received 7 July 1989; and in revised form 28 September 1989.

Our previous studies (Rodrigues *et al*., 1988) have shown that a significant increase in the phosphorous metabolite ratios occurs within 2 days of ovariectomy in rat NMU-induced mammary tumours, before any significant regression is evident. In the present study we have compared the histological changes in 20 similar tumours after ovariectomy or sham-ovariectomy with changes in the [31]P-NMR spectra of the tumours. Tumours were studied at 2 days post-operation, when ovariectomy had caused a significant NMR change but no regression, and at 14 days post-operation, when we had found both NMR changes and significant regression.

### Materials and methods

Oestrogen sensitive mammary tumours were induced in female virgin Ludwig/Wistar/Olac rats (Olac 1976 Limited, Oxon, UK) essentially by the method described in Williams *et al*. (1981). The animals were kept at 19°C in isolators with a photo period of 12 h per day. They were fed CRM diet (Labshore, Croydon, UK) and received water *ad libitum*. NMU (Sigma Chemical Co., Poole, Dorset, UK) was dissolved in distilled water at 12.5 mg $ml^{-1}$ and adjusted to pH 5.4 with acetic acid. Fifty-day-old rats were given three doses of NMU (50 mg $kg^{-1}$ body weight) at 2-weekly intervals. They were then transferred to our animal house where they were kept at 22-23°C with a 12 h light period per day and fed SDS diet (Special Diet Services Ltd, Witham, UK). After 20 weeks 80% of the animals developed mammary tumours. The tumour volume was measured using the following formula, where $d_1$, $d_2$, and $d_3$ are the length, width and depth of the tumour:

$$v = \pi/6\ (d_1.d_2.d_3)$$

### *NMR methods*

When the tumours had grown to 1.5-2 cm diameter the animals were anaesthetised with pentobarbitone (30 mg $kg^{-1}$ i.p.) and placed within the 27 cm bore of a 1.89 Tesla Oxford Research Systems TMR 32 200 NMR instrument. Spectra were obtained at 32 MHz using 1 or 1.4 cm diameter surface coils (Ackerman *et al*., 1980) and pulse durations of 6 or 8 $\mu$s respectively from the tumours, according to their volumes. In the regressing tumours, of which some had reached $<$40% of their initial volumes, the smaller coil was always used. The pulse repetition time was 3 s and 480 scans were collected routinely. Peak integrals of PCr and β NTP were calculated using the software package supplied with the instrument after its profile correction routine had been used to remove some of the broad signals. Due to difficulties in baseline definition and overlapping peaks these integrals are all expressed as ratios of integrals, which minimises some of the uncertainties. The mean and standard error of the mean are reported throughout and the significance of difference was tested by Student's $t$ test.

*Histological sections*

Of the tumours used in this study five from each of the four groups (i.e. 2 and 14 day post-sham operation or post-ovariectomy) were cut into 2–3 mm slices and fixed in modified methacarn (Mitchell *et al.*, 1985) before processing and paraffin embedding. Three representative haematoxylin and eosin stained sections were examined for tumour classification and tissue architecture.

Parallel sections were stained immunocytochemically with antibodies directed to antigens that delineate myoepithelial cells, luminal cells and basement membranes. The tumours were classified according to the new World Health Organization classification of rat mammary tumours (Russo *et al.*, 1989*a*) and as described in detail in the International Life Sciences Monograph (Russo *et al.*, 1989*b*).

*Mitotic index*

In each slide fields were selected at random using a × 40 objective. Fields in which epithelial cells occupied more than 50% of the area were included for counting of mitotic figures. The total number of epithelial cell nuclei and mitotic figures were counted in each field. Ten fields were screened on each slide and the mitotic index calculated. Some 3,000–6,000 cells were counted per tumour.

*Immunocytochemistry*

Wax-embedded 5 μm thick sections were de-waxed in xylene and then passed through a graded series of alcohols to water. Protease digestion when required involved a 15 min incubation at 37°C, with 4 mg 100 $ml^{-1}$ of pronase (Calbiochem-Behring) in phosphate buffered saline (PBS). Endogenous peroxidase was blocked by soaking the slides in 0.1% phenylhydrazine hydrochloride in PBS for 5 min at room temperature. Antibodies were made up in PBS (pH 7.4) containing 0.5% (w/v) bovine serum albumin (BSA; Sigma) and incubation was allowed to proceed for 1 h at room temperature for each antibody. An indirect peroxidase technique was used as previously described (Dubois *et al.*, 1987). The secondary antibody for the rabbit polyclonal antiserum was a peroxidase conjugated swine or goat anti-rabbit IgG (Dakopatts, High Wycombe, Bucks., UK) used at 1:25 dilution. The chromagen used was diaminobenzidine tetrahydrochloride (Sigma). All sections were counterstained with Meyer's haematoxylin. Non-specific staining by the secondary antibody was controlled by omitting the primary antibody. The specificity was confirmed by prior absorption of the antibody with purified antigen (1 mg $ml^{-1}$ for 1.5 h at room temperature) (Gusterson *et al.*, 1982).

*Antibodies*

*Keratin* This antiserum, which was raised in rabbits against human callus keratin, strongly stains normal breast myoepithelial cells in methacarn fixed paraffin embedded sections after protease digestion (Warburton *et al.*, 1982). The antiserum was used at a dilution of 1:100.

*Actin* This polyclonal rabbit antiserum was produced by inoculating rabbits with actin extracted and purified for denatured chicken gizzard actin (Bussolati *et al.*, 1980). This antiserum was used at a dilution of 1:100 and was a gift from Dr J. Couchman (Unilever Research, UK).

*Laminin* Antiserum to murine laminin have been previously described and was used at a dilution of 1:200. Prior protease treatment of the sections gave enhanced staining.

*Rat milk fat globule membrane* An antiserum raised in rabbits to the rat milk fat globule membrane (Warburton *et al.*, 1982) was used to delineate the luminal cells in the rat breast. The antiserum was used at a dilution of 1:200.

### Results and discussion

*NMR data*

NMR spectra were collected on each of 15 tumours the day before ovariectomy or on each of five tumours before sham operation took place. A representative spectrum taken the day before treatment is shown in Figure 1a and a spectrum from the same animal collected 14 days after ovariectomy in Figure 1b. The increase in the PCr signal relative to NTP can be clearly seen. No such effect was seen in the sham operated animals. There is the possibility that some of the NMR signal in subcutaneously implanted animal tumours comes from the overlying skin as well as from the tumours (Stubbs *et al.*, 1988, 1989). However, the proportion of signal coming from overlying tissues is dependent among other things on the size of animal and on the site of tumour growth (Stubbs *et al.*, 1989). These particular tumours have grown up in sites associated with mammary tissue, i.e. ventral, and in relatively small rats (200–230 g) where the contribution from skin is minimal. A control experiment where a glass spherical phantom was implanted into the site of an excised regressing tumour also suggested there was no significant contribution from surrounding tissues.

Because of the heterogeneity of chemically induced tumours we have analysed the NMR spectra comparing the 2 and 14 day post-ovariectomy or sham data with their own controls (i.e. spectra taken 1 day before operation, see Table I). Before ovariectomy the PCr/βNTP ratios in the treated groups and in the smaller sham-operated group were very similar (Table I). Two days after ovariectomy the value of PCr/βNTP had increased significantly in the animals that had undergone ovariectomy ($P<0.02$) and after 14 days PCr/βNTP was more than twice the mean ratio before operation ($P<0.001$). However, no significant changes occurred after sham operation ($P>0.1$) at either 2 or 14 days. No

**Figure 1** The effect of ovariectomy on the $^{31}$P-NMR spectra of an NMU induced mammary adenocarcinoma. **a**, Spectrum collected the day before ovariectomy. **b**, Spectrum collected 14 days after ovariectomy. Peak assignments as follows: 1, β-phosphate of NTP; 2, α-phosphate of NTP, α-phosphate of ADP, NAD; 3, γ-phosphate of NTP, β-phosphate of ADP; 4, phosphocreatine; 5, phosphodiester; 6, Pi; 7, phosphomonoester.

**Table I** Effect of ovariectomy on PCr/βNTP measured by NMR

| *Days before or after ovariectomy/ sham operation* | *PCr/βNTP ovariectomy* | | *PCr/βNTP sham operation* | |
|---|---|---|---|---|
| 1 day before | 0.54 ± 0.06 | 0.74 ± 0.098 | 0.49 ± 0.33 | 0.60 ± 0.25 |
| 2 days after | 1.04 ± 0.20* | – | 0.41 ± 0.094 | – |
| 14 days after | – | 1.81 ± 0.19** | – | 0.44 ± 0.03 |

The ratios were calculated from integrals of peak areas from the NMR spectra. Because of the heterogeneity of chemically induced tumours the results are expressed as mean ± s.e.m. at each time point compared to the appropriate control group, i.e. 1 day before operation result. There were 15–16 animals in each of the ovariectomy groups and 3–4 animals in the sham groups. *$P<0.02$, **$P<0.001$ compared to 1 day before.

significant change in tumour pH, either in the sham or ovariectomised groups ($P>0.1$) was observed.

### *Changes in tumour volume*

Ten tumours from the sham group and 10 from the ovariectomy group were taken for histology. The volumes of these tumours were $6.06 \pm 0.76\ cm^3$ ($n = 10$) in the group selected for sham operations and $4.44 \pm 0.77\ cm^3$ ($n = 10$) in the group selected for ovariectomy. The expected decrease in volume was seen 14 days after ovariectomy (41 ± 13% of the volume on day before operation; $n = 5$, $P<0.05$). No significant change ($P>0.1$) was observed at 2 days in either the ovariectomy group (113 ± 26%, $n = 3$) or at either 2 days (125 ± 14%, $n = 4$) or 14 days (109 ± 14%, $n = 4$) in the sham operated controls.

### *Histology*

Using the new WHO classification (Russo *et al.*, 1989*a*,*b*) the majority of tumours induced by NMU and other carcinogens are classified as adenocarcinomas. Although they very rarely show evidence of metastatic spread, the criteria for malignancy are based upon cytological abnormalities and local growth pattern. The majority of the tumours in this study are adenocarcinomas of the cribriform/papillary type, with a predominance of papillary pattern. As in other studies, it should be stressed that the growth pattern of these lesions varies in different parts of the tumour. In the haematoxylin/eosin sections, however, regardless of the growth pattern, there appear to be two cell populations present within the epithelial component of these tumours. Adjacent to the stroma on the outer aspect of the tumour islands, there is a morphologically different cell population which is a presumptive myoepithelial phenotype and which is often difficult to see on routinely stained preparations (Figure 2a). Inside this is another cell population of distinct phenotype. In some areas it is single layered, but in most areas consists of multilayers of cells with a large amount of cytoplasm, some of which shows vacuolation suggesting secretory activity. The solid areas of epithelial cells are separated by intervening strands of connective tissue containing numerous mast cells. The histological sections of the tumours showed that four of the specimens examined were not all adenocarcinoma (see Table II). One of these was a fibroadenoma and another consisted predominantly of normal lymph nodes (numbers 9 and 12 respectively). Two other tumours, numbers 4 and 15, were macroscopically thought to be adenocarcinomas; one of them, number 4, showed a small focus of adenocarcinoma but the majority of the lesion consisted of fibrosis and a small focus of adenoma. The other, number 15, showed a classical fibrosarcoma.

### *Two-day and 14-day post-sham operated rats*

The majority of the tumours showed typical NMU induced adenocarcinomas of the cribriform/papillary type. One

**Table II** Combined NMR and histology study of ovariectomy in NMU-induced mammary tumours

| *No.* | *% size change* | *PCr/βNTP* | *Histology* |
|---|---|---|---|
| *Sham ovariectomy, 2 days* | | | |
| 2 | 104 | 0.59 | cribriform/papillary adenocarcinoma |
| 5 | 100 | 0.31 | cribriform/papillary adenocarcinoma |
| 10 | 155 | 0.50 | cribriform/papillary adenocarcinoma, 50% carcinosarcoma |
| 15 | 200 | 0.57 | all fibrosarcoma |
| 18 | 143 | 0.09 | cribriform/papillary adenocarcinoma |
| *Sham ovariectomy, 14 days* | | | |
| 4 | Regressed | 0.44 | small area of adenocarcinoma of papillary type/tubular adenoma/fibrosis |
| 6 | 125 | Poor spectrum | cribriform/papillary adenocarcinoma |
| 11 | 139 | 0 (PCr not detectable) | cribriform/papillary adenocarcinoma |
| 13 | 82 | 0.39 | cribriform/papillary adenocarcinoma |
| 20 | 91 | 0.50 | cribriform/papillary adenocarcinoma, 50% dense, fibrous mass |
| *Ovariectomy, 2 days* | | | |
| 1 | – | 2.56 | cribriform/papillary adenocarcinoma |
| 7 | 72 | 0.63 | cribriform adenocarcinoma |
| 9 | 125 | Poor spectrum | Dense collagen (fibroadenoma) |
| 16 | 160 | 1.66 | cribriform/papillary adenocarcinoma |
| 17 | 106 | Poor spectrum | cribriform/papillary adenocarcinoma |
| *Ovariectomy, 14 days* | | | |
| 3 | 34 | 1.56 | 90% tubular adenoma |
| 8 | 38 | Poor spectrum | 90% tubular adenoma pattern. Focal adenocarcinoma |
| 12 | 4 | Poor spectrum | 25% tumour, rest lymph node |
| 14 | 78 | 2.17 | tubular adenoma pattern |
| 19 | 13 | 3.10 | tubular adenoma pattern. Focal adenocarcinoma |

tumour (number 10) had a biphasic growth pattern, being 50% adenocarcinoma and 50% carcinosarcoma. Another (number 15) was composed of malignant spindle cells and classified as a fibrosarcoma. This tumour was eliminated from the NMR study. (See Table II.)

Within the typical adenocarcinomas both antibodies to actin and keratin (Figure 2b) defined a fragmented but very definite myoepithelial layer. This myoepithelial population was adjacent to a clearly defined basal lamina as demonstrated with the anti-laminin antibody (Figure 2c). Within the tumours the luminal cell population was usually stratified with a high mitotic activity (0.20 ± 0.061%, $n = 5$ and 0.136 ± 0.052%, $n = 5$ in the 2- and 14-day sham operated respectively) and prominent nucleoli. In some areas, where very clearly defined luminal structures were formed, the myoepithelial layer was very prominent (Figure 2b).

### *Two days post-ovariectomy*

In four of the tumours (number 9 was eliminated from the study as it was a fibroadenoma) typical cribriform/papillary adenocarcinoma was present with focal necrosis. There were no features seen on either the haematoxylin/eosin stained

**Figure 2** **a**, Photomicrograph of a typical adenocarcinoma composed of a multi-layered luminal cell population with a high mitotic activity. Magnification × 312.5. **b**, Adencarcinoma stained with keratin showing differentiation of myoepithelial cells at the epithelial-stromal junction. Magnification × 312.5. **c**, Adencarcinoma stained with a polyclonal anti-laminin antiserum demonstrating a clearly defined basal lamina at the epithelial-stromal junction. Magnification × 312.5. **d**, Photomicrograph of an adenocarcinoma composed of a multi-layered luminal cell population 2 days after ovariectomy. Magnification × 312.5. **e**, Typical tubular adenoma-like pattern seen 14 days post-ovariectomy. On this haematoxylin and eosin section the lumina now appear to have only one cell layer and there is an increase in the nuclear to cytoplasmic ratio compared with the adenocarcinomas. Magnification × 312.5. **f**, A tumour 14 days post-ovariectomy stained with the antikeratin antibody shows attenuated myoepithelial cells at the periphery (arrows). Magnification × 350. **g**, 14 days post-ovariectomy tumour stained with anti-laminin shows diffuse increase in basement membrane material between the glandular elements. Magnification × 350.

sections or using immunocytochemistry, which could be attributed to the ovariectomy either in cytological characteristics or in overall architecture (see Figure 2d). However, the mitotic index at 0.076±0.039% ($n = 5$) was decreased compared to the 2-day sham group (0.20±0.061%) although this difference was not significant ($P > 0.1$).

*Fourteen days post-ovariectomy*

There was a striking change in the morphology of these tumours after ovariectomy, all of them being indistinguishable from typical tubular adenomas (Figure 2e). The mitotic index was significantly different from the 14-day sham operated animals at 0.008±0.005% ($P < 0.05$). Two of the tumours, numbers 8 and 19, showed small foci of residual adenocarcinoma which made up less than 10% of the tumour as assessed microscopically. Within the areas of adenocarcinoma, there was evidence of central degeneration of the multilayered structures to leave an apparent single layer of viable cells forming small lumina. Within the tubular adenoma-like areas it was often difficult to identify two cell layers as the myoepithelial cells were very flattened and attenuated and could often only be identified with antibodies to actin and keratin (Figure 2f). The luminal cell population as defined with the antibodies to milk fat globule membrane were of a single layer of low cuboidal cells with a higher nuclear to cytoplasmic ratio than that seen in adenocarcinomas. Mitotic activity was also significantly reduced in the adenoma-like areas compared with the adenocarcinomas. With the reduction in the luminal epithelial component of these tumours, there was a relative increase in proportion of myoepithelial cells and in the amount of connective tissue. The spaces now separating the glandular elements were filled with an apparent increase in basement membrane material as demonstrated with the antibodies to laminin (Figure 2g). This was a very striking feature in all of the tumours.

The changes described here in relation to ovariectomy are similar to those that were previously reported by Young and Hallowes (1976) and more recently by Lancaster *et al.* (1988). A number of points should be noted. These tumours are heterogeneous in terms of the two cell populations which are present. This is unlike human breast carcinomas, where the current evidence strongly suggests that they are derived from the luminal cell population (Gusterson *et al.*, 1982). It is therefore essential to distinguish between antigenic heterogeneity, as seen in human breast cancer, and heterogeneity of the type seen in rat tumours where there are two cell populations. This raises questions concerning the relevance of the rat model for certain biological studies although the model is very useful to assess hormone responsiveness, whether due to ovariectomy or chemical manipulation. There are a number of changes in the tumours which can be associated with ovariectomy. These are a reduction in the hyperplastic nature of the malignant luminal cell population and cytological differences in this population post-ovariectomy which are caused by a reduction in the amount of cytoplasm. The loss of cytoplasmic ratio after ovariectomy is due to a change in the secretory activity of these cells. There is also an obvious reduction in the number of mitotic figures seen after ovariectomy. The striking increase in basement membrane material is an interesting observation which is worthy of further study.

The identification microscopically of tumours other than adenocarcinomas indicates the importance of histological examination in any studies where correlations between tumour behaviour and treatment are being considered, especially in chemically induced tumours. The absence of histological changes in the tumours at 2 days post-ovariectomy (when no detectable regression had occurred) suggests that the early NMR changes are due to alterations in the metabolism of the tumour cells. This is consistent with a hypothesis that we have previously put forward (Rodrigues *et al.*, 1988). The steady fall in the high energy phosphate (i.e. PCr and NTP) peaks in the spectrum of an untreated tumour suggests that these tumours outgrow their blood supply, a common phenomenon in animal tumours. When oestrogens are withdrawn from an oestrogen-dependent mammary tumour, cellular growth ceases and the tumour's requirements for oxygen and other nutrients are greatly reduced. Removing the drive to growth would allow the cellular energy reserves to be repleted and thus lead to the paradoxical improvement in the high energy phosphate status of a tumour that is about to regress. These metabolic changes probably also reflect the early events related to the decrease in mitoses and luminal cell population and the increase in basement membrane material observed at 14 days post-ovariectomy.

We would like to acknowledge the technical assistance of Mrs Shona Harrison, Mr G. Harrison and Ms Pritti Shah. We thank Dr Warburton for the kind gift of the antibodies to keratin, laminin and milk fat globule membrane and Dr Anbazhagan for assistance with the histology. Supported by the Cancer Research Campaign, UK (M.S., R.C.C., J.R.G., R.J.M., L.M.R., B.A.G.) and the Medical Research Council (B.A.G.).

### References

ACKERMAN, J.J.H., GROVE, T.H., WONG, G.G., GADIAN, D. & RADDA, G.K. (1980). Mapping of metabolites in whole animals by 31P NMR using surface coils. *Nature*, **283**, 167.

BUSSOLATI, G., AL FARI, V., WEBER, K. & OSBORNE, M. (1980). Immunocytochemical detection of actin on fixed and embedded tissues. Its potential use in routine pathology. *J. Histochem. Cytochem.*, **28**, 169.

DUBOIS, J.-D., O'HARE, M.J., MONAGHAN, P., BARTEK, J., NORRIS, R. & GUSTERSON, B.A. (1987). Human breast epithelial xenografts: an immunocytochemical and ultrastructural study of differentiation and lactogenic response. *Differentiation*, **35**, 72.

EVANOCHKO, W.T., NG, T.C. & GLICKSON, J.D. (1984). Application of *in vivo* NMR Spectroscopy to cancer. *Magn. Reson. Med.*, **1**, 508.

GUSTERSON, B.A., WARBURTON, M.J., MITCHELL, D., ELLISON, M., NEVILLE, A.M. & RUDLAND, P.S. (1982). Distribution of myoepithelial cells and basement membrane proteins in the normal breast and in benign and malignant breast diseases. *Cancer Res.*, **42**, 4763.

LANCASTER, S., ENGLISH, H.F., DEMERS, L.M. & MANNIA, A. (1988). Kinetic and morphometric responses of heterogeneous populations of experimental breast cancer cells *in vivo*. *Cancer Res.*, **48**, 3276.

MITCHELL, D., IBRAHIM, S. & GUSTERSON, B.A. (1985). Improved immunohistochemical localisation of tissue antigens using modified methacarn fixation. *J. Histochem. Cytochem.*, **33**, 491.

NG, T.C., EVANOCHKO, W.T., HIRAMOTO, R.N. & 6 others (1982). 31-P NMR spectroscopy of *in vivo* tumours. *J Magn. Reson.*, **49**, 271.

RODRIGUES, L.M., MIDWOOD, C.J., COOMBES, R.C., STEVENS, A.N., STUBBS, M. & GRIFFITHS, J.R. (1988). 31-P Nuclear magnetic resonance spectroscopy studies of the response of rat mammary tumours to endocrine therapy. *Cancer Res.*, **48**, 89.

RUSSO, J., RUSSO, I.H., ROGERS, A.E., VAN ZWIETEN, M.J. & GUSTERSON, B.A. (1989*a*). Classification of neoplastic and non-neoplastic lesions of the rat mammary gland. In *Integument and Mammary Glands*, Jones, T.C., Mohr, U. & Hunt, R.D. (eds) p. 275. Springer Verlag, Berlin.

RUSSO, J., RUSSO, I.H., ROGERS, A.E., VAN ZWIETEN, M.J. & GUSTERSON, B. (1989*b*). In *Pathology of Tumours in Laboratory Animals*, 2nd edition, Turosov, V. & Mohr, U. (eds). IARC: Lyon.

STEEN, R.G., TAMARGO, R.J., MCGOVERN, K.A. & 4 others (1988). *In vivo* 31P NMR spectroscopy of subcutaneous 9L gliosarcoma: effects of tumour growth and treatment with 1,3-*Bis* (2-chlorethyl)-1-nitrozurea on tumour bioenergetics and histology. *Cancer Res.*, **48**, 676.

STUBBS, M., RODRIGUES, L.M. & GRIFFITHS, J.R. (1989). Potential artefacts from overlying tissues in $^{31}$P-NMR spectra of subcutaneously implanted rat tumours. *NMR Biomed.*, **1**, 165.

STUBBS, M., VANSTAPEL, F., RODRIGUES, L.M. & GRIFFITHS, J.R. (1988). Phosphate metabolities in rat skin. *NMR Biomed.*, **1**, 50.

TOZER, G.M., BHUJWALLA, Z.M., GRIFFITHS, J.R. & MAXWELL, R.J. (1989). Phosphorus-31 magnetic resonance spectroscopy and blood perfusion of the RIF-1 tumour following X-irradiation. *Int. J. Radiat. Oncol. Biol. Phys.*, **16**, 155.

WARBURTON, M.J., MITCHELL, D., ORMEROD, E.J. & RUDLAND, P.S. (1982). Distribution of myoepithelial cells and basement membrane proteins in the resting, pregnant, lactating and involuting rat mammary gland. *J. Histochem. Cytochem.*, **30**, 667.

WILLIAMS, J.C., GUSTERSON, B., HUMPHREYS, J. & 4 others (1981). *N*-methyl-*N*-nitrosourea-induced rat mammary tumours. Hormone responsiveness but lack of spontaneous metastasis. *J. Natl Cancer Inst.*, **66**, 147.

YOUNG, S., & HALLOWES, R.C. (1976). Tumours of the mammary gland. In *Pathology of Tumours in Laboratory Animals, vol.1*, Turosov, V.S. (ed) p. 31. IARC: Lyon.